# Murchison on Fever
**A Treatise on the Continued Fevers of Great Britain.* By Charles Murchison, M.D., F.R.C.P., Senior Physician to the London Fever Hospital, &c. 8vo, pp. 638. London, 1862.


**Published:** 1863-10

**Authors:** 


### Art. X.—MURCHISON ON FEVER.*

"In the whole range of human maladies there is no disease," Dr. Graves has said, "of such surpassing interest and importance as fever." Whether it be regarded in its bed-side or in its social aspect—whether as a question of practical medicine or of public hygiene, it compels a degree of attention exceeding that

* *A Treatise on the Continued Fevers of Great Britain.* By Charles Murchison, M.D., F.R.C.P., Senior Physician to the London Fever Hospital, &c. 8vo, pp. 638. London, 1862.

of any other affection. No malady exacts so much from the resources of the physician, none so fully displays the power of the healing art. No malady so deeply concerns the welfare of the public—none more narrowly affects the vigour and prosperity of the community—none more closely links the physician's duty with the national good.

If fever (restricting here the term to continued fever) be regarded as a cause of vital waste, but one acute disease (pneumonia) exceeds it in fatality; if as a source of permanent loss of physical vigour, a drag upon the productiveness and well-being of a people, none can be esteemed more potent. It sweeps off more than seventeen thousand lives annually in England and Wales alone, the major portion in the dawn of life, the spring of youth, and the flush of manhood. One-third of this great loss occurs within the first ten years of life; another third from the tenth to the thirty-fifth year; and the mortality is greater the nearer the outset of life is approached. One-fifth of the whole loss falls in the first lustrum—in infancy and early childhood.*

But death is one part only, although the greatest, of the evil which fever inflicts upon a community. To the irreparable loss which arises from the premature removal of so great a proportion of its most valuable lives, is to be added the permanent loss of vigour arising from the large amount of constant sickness originating in the same cause. This grave drawback falling chiefly upon the bread-winners of the poorer classes, is one of the most important sources of impoverishment and pauperism, and it imposes a heavy burden upon the charitable means of the country. When fever prevails epidemically, the truth here stated is conspicuous to the least observant; but it is too frequently forgotten in ordinary periods. Fever, however, is at all times present among the community, and it cannot be too carefully borne in mind that the accumulated mischief the malady creates in the intervals of epidemics, far exceeds that which arises while an epidemic is present. The proportion of persons attacked with the disease is estimated by Dr. Murchison as ten times greater than the mortality from it. During the last twenty years no less than 350,000 of the population of this country have been destroyed by fever. This number of deaths would, according to the foregoing estimate, represent 3,500,000 cases of the malady within the same period. Add the days and weeks of disabling sickness, and an imperfect notion maybe formed of the magnitude of the subject in its relation to the well-being of a community.

* See Table of Deaths from Continued Fever, for the ten years 1851-60, in the *Registrar-General's* 24*th Annual Report*, p. 217.

Again; fever has often assumed the dimensions of a national disaster, and the history of the disease includes some of the greatest calamities which have befallen mankind. Twice within the past twenty years England has experienced this sad truth: first, in the pestilential outbreak which accompanied the Irish famine of 1847; secondly, in the catastrophe which befell the British army, during the winter of 1854, in the Crimea. It has been asserted, and not without some degree of probability, that the enormous losses which the French army suffered from fever before Sebastopol, in the winter of 1855, when the camps and hospitals of our allies were ravaged with typhus, exercised an important influence in bringing the Russian War to a close. Throughout the winter of last year, England watched with dread the fore-warnings of pestilential fever among the famine-stricken cotton-workers of Lancashire, but the danger was happily averted.

The medical aspect of the subject is commensurate in magnitude with its social. At all times commanding the attention of the physician, fever holds a no less prominent place in the history of medicine than it does in the history of peoples. But so great and complicated are the questions involved in the study of the disease, that the growth of a just knowledge of the conditions under which it is developed, and of its true character, would seem to be but of yesterday. If we have mastered the all-important truth, itself of recent date, that the great disability and destruction of life from fever is avoidable, we have hardly yet learned to distinguish under one and the same term, essentially different forms of disease, and this source of confusion still hampers our systematic treatises and our mortuary records. We have still to learn that under the same apparent conditions of development specifically different morbific agencies may exist. We occupy, in short, a transition period, in which we are compelled to retain much in ideas and phraseology that is old, and yet may not set aside or neglect the new. Mr. Simon, writing in 1858, has most aptly described this period. His description forms so excellent a stand-point from which to estimate the value of Dr. Murchison's work, that we shall not hesitate to quote it in detail.

"The common judgment of the medical profession," he says, "on the controllability of continued fever is well expressed in a phrase which the late M. Baudens, an eminent physician of the French army, used in describing his Crimean experience of the disease—*On pourrait le faire naître et mourir à volonté.*

"It is essentially a disease of filth. Where the unventilated atmosphere of habitually overcrowded places reeks with a stagnant steam from the breathing and sweating of its inhabitants —a steam which condenses in fœtid drops on the window-panes, or soaks and rots in the papered or plastered walls; or where putrefying fæces are accumulated in cesspools or ill-conditioned drains, to taint the air or leak into the drinking water of a population; there this disease prevails, in one or other of its forms.

"*In one or other of its forms*, I say—for the researches of modern pathologists have shown that, for accuracy's sake, it is requisite to distinguish at least two forms of continued fevers. And it seems highly probable that these forms, while being equally associated with filth, are yet not both essentially associated with the same kind of filth. One of them (the *typhoid fever* of modern observers) has intimate affinity to the cause last mentioned—the fæcal pollution of air and water. The other, (which is now distinctively called *typhus*) more nearly associates itself with overcrowding, especially of destitute persons, and probably has its essential source in the putrefaction of the undispersed exhalations. The typhoid form, specially affecting the intestinal canal, is, in its nature as in its causes, very closely related to the diarrhœal diseases already spoken of. There exists no conclusive evidence to show whether this form of disease be in any degree or manner contagious; but almost certainly it cannot spread atmospherically by means of exhalations from the sick. Distinctive typhus, on the other hand, works its chief results without affecting the bowels. Possibly its first and greatest influence is exerted on the blood, but its symptoms are chiefly obvious in the nervous system, the skin, and the lungs, and the exhalations from a patient undergoing it are, till they have been neutralized by dilution with pure air, capable of communicating the same form of disease. It has some hitherto unexplained connexion with extremes of poverty and destitution. No such ravages have been made by it as when it has been associated with famine, and, apparently by reason of the association, has prevailed as a national epidemic.

"A knowledge of the distinction between these two forms of disease has hitherto not become general enough in England for the name of typhoid fever to have been kept separate in the registration returns. Though probably more fatal in ordinary years than true typhus, with which it is confounded, it has hitherto no statistical existence. I have, therefore, no choice but to speak of continued fever as though it were but a single form of disease, communicable from person to person."*

Now Dr. Murchison, in the treatise we have under conside-

---

* *Papers relating to the Sanitary State of the People of England*, p. 15. (Blue Book.)

ration, starting from the point of view here indicated, aims to set forth in a systematic form the progress which of late years has been made in our knowledge of continued fever. Such a work, duly executed, should reduce to order the confusion of the transition state between the new and the old, should bridge over the great gap between existing treatises and the actual state of our knowledge, and, while itself advancing, should furnish a solid foundation for the further advance of this knowledge. These are, indeed, the great features of Dr. Murchison's work. Bringing to his task a large experience of the subject of which he treats, rare erudition, and a matured judgment, this treatise at once takes its place among English medical classics. Advocating the doctrine of the plurality of continued fevers, he supports it on etiological as well as semeiological grounds. The prominence which Dr. Murchison gives to the etiology of these disorders is one of the most noteworthy characters of his treatise. For the first time this subject is given that development in a systematic treatise which a just apprecia-tion of its importance in reference to public and private hygiène demands. It was high time that the virtual divorce between the preventive and curative medicine of fever in our current text-books, was brought to an end, and Dr. Murchison has happily effected this.

Dr. Murchison adopts the following classification of con-tinued fevers :—

| | | | |
|---|---|---|---|
| A.—Non-Specific. | I.—Simple Fever, caused by . . | | Exposure to sun, surfeit, &c. |
| B.—Specific | II.—Endemic (Pythogenic, Enteric, or Typhoid,) caused by | | Poisons contained in emanations from sewers, &c. |
| | III. & IV. | Typhus, caused by | The concentrated exhalation, from squalid human beings. |
| | | Relapsing Fever . | Famine. |

In an introductory chapter Dr. Murchison treats briefly of the importance of the study of continued fevers, their classifica-tion, different species, the prevention of their causes, their spontaneous origin, the theory of pyrexia, and indications of treatment. In subsequent chapters he examines systematically the different forms of continued fever—including elaborate and most valuable accounts of their nomenclature and of their history. The importance of the detailed nomenclature in the study of the older writers cannot well be overrated. In con-cluding chapters he examines the circumstances influencing the mortality of continued fevers at different places, and the relative merits of isolating patients suffering from infectious fevers, and of distributing them in the wards of a general hospital. A copious bibliography and excellent index are appended to the work; which is also embellished with diagrams, woodcuts, and several exquisitely coloured lithographs of the eruptions observed in typhus and typhoid fever from admirable drawings by Dr. Westmacott.

In the subsequent remarks, we propose chiefly to give a brief sketch of the etiology of the three principal forms of continued fever, dwelling more particularly on the differential etiology of typhus and typhoid.

The excellent researches of Dr. Jenner furnished the finishing link in the chain of evidence which was required to establish the non-identity or specific difference of these two forms of fever. Since the publication of his inquiries we think no unprejudiced mind can any longer refuse to acknowledge that typhus and typhoid fever are as distinct from one another as scarlatina and measles. The existence of the peculiar lesions in the solitary and agminated glands, found by Bretonneau and Louis, in cases of continued fever in France, led to a series of observations on the *post-mortem* appearances of the fever which prevailed in Great Britain and Ireland, shortly after the time that their researches were made known in this country. Now this happened to be typhus; the changes anticipated were not found, and hence, at first, they were held to be merely an accident in the variety of the disease, which was common in Paris. More extensive research, however, led to the conclusion that, in many cases presenting altogether a different type, these changes were present; and by a careful juxtaposition of the various symptoms during life with the appearances found after death, it was made evident that two distinct forms of fever, differing in many most essential particulars, had been confounded together. The distinction of typhus and typhoid fever was subsequently admitted; and although some still refuse to acknowledge that they are not essentially the same disease, practically they are generally distinguished.

Typhus is, for the most part, a disease of adult age; typhoid of youth and adolescence. From the observation of a large number of cases of both diseases, admitted into the London Fever Hospital, 3456 cases of typhus and 1772 cases of typhoid, it would appear that persons over thirty years of age are twice as liable to typhoid fever as persons under that age. While on the question of age, we may note the rate of mortality increases rapidly in typhus with increasing age; but in typhoid, although somewhat greater in old persons, there is not that marked increase which occurs in typhus.

The greater number of cases of typhoid occur during the autumn. Typhus does not seem influenced, save indirectly, by the seasons; if epidemic, it does not become more prevalent with the commencement of cold weather, nor does it decline on the advent of summer; its increased frequency in the latter half of a prolonged cold winter, and the commencement of spring, is probably due to the overcrowding and bad ventilation of the dwellings of the poor at such periods, and the increased predisposition which is often induced by the debility, the result of long-continued destitution, so frequent towards the end of an inclement season.

Typhoid fever is essentially an endemic disease; typhus, epidemic. Typhoid has formed no part of any great fever epidemic; it may become epidemic at times in localities where it was previously unknown, but it is then local and circumscribed, and can generally be traced to a definite cause. It is confined to a single house or village, and does not extend beyond certain limits; on this account it is frequently named after the localities in which it occurs, as the "Croydon fever," the "Windsor fever," &c. Destitution, starvation, and overcrowding constitute the most powerful predisposing causes of typhus; it is essentially the fever of the poor. These three conditions are so generally combined, that the amount of influence exercised by famine and overcrowding, separately, can scarcely be appreciated. Under this head Dr. Murchison remarks:—

"A careful study of the records of typhus epidemics demonstrates, in my opinion, the intimate connexion between these epidemics and famine or distress. They have appeared during every variety of climate, season, and weather. Famine and overcrowding have been the sole conditions common to them all. In fact, on more than one occasion, epidemics have been predicted from the occurrence of famine; and the result has verified the prediction.

"Some persons imagine that famine from failure of the crops, and epidemics of typhus, both result from one common cause, such as an obscure 'atmospheric,' or 'epidemic' influence. But against such a view it may be argued:—First, that in bodies of men living in the same locality, and exposed to the same atmospheric influences, the prevalence of typhus has been found to be in a direct ratio to the degree of privation. . . . Secondly, epidemics of typhus appear during the state of privation consequent on strikes, commercial failure, and warfare; or, in other words, artificially induced famine entails the same results as the famine arising from the failure of the crops." (p. 77.)

Famine produces a state of weakness of the system which renders it peculiarly liable to an attack of typhus, if typhus should present itself; but "famine only generates typhus in so far as it causes overcrowding."

Typhoid fever, on the other hand, is by no means a disease of poverty and destitution; indeed, it is the upper and middle classes that constitute the majority of its victims.

The proximate cause of typhus is a specific poison known as the typhus poison—the product of the disease itself—or, at times, generated *de novo*. That typhus fever is contagious, is so generally admitted, that it is needless now to recapitulate the evidence which has been produced to prove it. The following facts, adduced by Dr. Murchison as to its contagion at the London Fever Hospital, may be new to some of our readers:—From 1848 to 1862, fourteen years and a half, eighty cases of typhus originated in the hospital; of these, forty-four were nurses, five medical attendants, three servants, and twenty-eight patients admitted with other diseases. The average number of medical attendants and nurses is about eighteen. Only three of the servants not engaged in the wards had the fever, and one of these had to receive and wash the patients on admission.

A few remarks as to some of the laws which regulate its contagion. It is seldom communicated to any great distance. The old London Fever Hospital was situated in the midst of a most crowded neighbourhood, and yet no instance was ever known of typhus fever spreading to any of the adjoining houses. The degree of concentration of the poison seems also to affect its contagious properties; a single patient, in a large, well ventilated room, seldom communicates the disease to any other inhabitant of the same house—rarely even to his immediate attendants. It can be communicated, also, by fomites, by articles of clothing, or by the furniture of an apartment; but, in order to do this, it is necessary that the poison should be very concentrated, and the room unventilated, and long exposed to its action. Several instances have been related of its transmission by infected ships, long after all the cases of fever had been removed. In the Crimean war, one of the French transports with passengers suffering from this fever, disembarked them all at Constantinople, and took on board other passengers uninfected; yet the fever broke out among them before they reached their destination. Woollen materials seem particularly to imbibe and communicate the poison; but if freely exposed to the air, and well ventilated, they soon lose the traces of contagion. No instance has been recorded of a medical man conveying typhus in his clothes from one patient to another. Exposure of the garments for some time to high temperature, seems to be the most convenient method of getting rid of the contagion from the clothes of typhus patients themselves.

Typhus has doubtless been the cause of some of the most terrible pestilences, both in civil and military life. It is probably a disease of great antiquity; and has certainly prevailed in Europe, at various times, for the last four centuries. The history of this fever is a subject of much interest to the medical historian; but in consequence of the somewhat indiscriminate and indefinite use of the term fever in the descriptions left us by the olden writers, and notwithstanding the amount of attention which has been given to this branch of the subject, it still remains in rather a confused state. Even in comparatively modern times, the different uses of the words typhus and typhoid—by some authors, as words of similar import, if not as identical—by others, with a sense of distinction more or less complete—by others, as indicating two perfectly distinct and separate diseases—have rendered the name given in the description of any particular epidemic of fever quite insufficient alone to establish its identity. On this matter the warning of Bacon recommends itself strongly to the attention of medical authors. "Certain it is," says he, "that words, as a Tartar's bow, do shoot back upon the understanding of the wisest, and mightily entangle and pervert the judgment: so that it is almost necessary in all controversies and disputations to imitate the wisdom of the mathematicians—in setting down in the very beginning the definitions of our words and terms—that others may know how we accept and understand them, and whether they concur with us or no. For it cometh to pass for want of this, that we are sure to end there where we ought to have begun — which is in questions and differences about words."

To the head of typhus may probably be referred most of the epidemics of *jail fever* in this country; and its communication from prisoners on trial, in the crowded and ill-ventilated courts, to the numerous persons obliged to be present on such occasions, forms the leading feature in those trials known in history as "Black Assizes." It was probably the same fever that was found by the great philanthropist Howard raging, in so many instances, among the ill-fed inmates of the overcrowded, ill-ventilated prisons.

As the *ship fever*, it has proved fatal to many a crew; especially in transport ships where numerous persons were crowded in a small space, and prevented by stormy weather from being much upon deck.

As the *military* or *camp fever*, its ravages have been more extended, and it has had more victims than in any other form. In besieged cities, and in the camp of besieging armies, especially in long continued and hotly contested sieges, it has proved often more fatal to both parties than the fire and the sword. There has been hardly a European campaign of any moment in which it has not sooner or later prevailed among the troops, from the time of the Thirty Years' War to the wars of the first Napoleon, and in our own days among the armies of his nephew. Among the French, the English, and the Russian troops, it has waged a discriminating warfare, regardless indeed of party, but attacking always with especial virulence the ill-fed, the weak, and those living in a crowded camp, and exposed to its poison in its most concentrated form. This leads us to remark that, although usually spread by contagion, it can, under certain circumstances, originate *de novo*.

Overcrowding, bad ventilation, and debility produced by a scanty supply of nourishment, especially if these conditions be of long duration and are accompanied with much mental depression, strongly favour its origin. Among the most recent instances of its development, is the outbreak of typhus which took place in the armies of the Crimea; and we quote these experiences here for the double purpose of showing the nature of the circumstances attendant on such an outbreak, and as a proof of the effect of proper hygienic measures in checking its extension and hindering its development. The large quantity of typhus, and the great mortality therefrom among the English troops when they were badly lodged, ill-fed, and crowded together in their tents, have now become a matter of history. This was in 1854–5, at the beginning of the campaign, when the state of the commissariat and the lodgment of the English troops formed such a striking contrast to those of the French. The mortality of the French from the same cause at this period was very small indeed. In 1856, however, the positions of the two armies were so reversed, that the French lost 6000 men from this fever, while the loss of the English was but trifling. We translate the description of the condition of the French army at the time of the prevalence of the fever in their camp, quoted by Dr. Murchison in his section on military fever:—"After a prolonged sojourn in the mud of the trenches—after being on guard, toiling at the field-works, and discharging extra duties of various kinds—after marching over roads like newly ploughed fields, and being drenched to the skin with rain and with snow—the soldiers, shivering with cold, and often not possessing a change of clothes, crowded together in their tents and huts, lighted, if possible, a scanty fire, and hermetically closed all the openings with a perseverance and constancy which the most urgent recommendations and the most stringent regulations in vain attempted to overcome. The extreme dirtiness of the men, their fœtid breath, the smoke of tobacco, the evaporation of the water from their saturated garments, all combined to empoison these narrow holes. Within was typhus; without the biting frost, sufficiently severe to produce gangrene of the extremities. Danger was everywhere, but the greatest danger was certainly within; the overcrowding was universal, even in the ambulance, constructed to hold two or three hundred men, three times that number were often accumulated." The medical inspector of the French army, M. Baudens, remarks: "The causes of typhus are so well known, that we can at our will create or destroy the conditions which produce it. Here the causes of its existence were doubtless the concentration and overcrowding of the men, induced by the rigour of the climate; crowded together, and hermetically sealed up in their tents, they fell easy victims to the poisonous miasmata." M. Jacquot, and the medical inspector of the Russian army, expressed the same opinion. At the same time, side by side of this suffering, the English army, now provided with large, well-ventilated tents, well clothed and well fed, otherwise in exactly similar circumstances with their allies, enjoyed almost complete immunity from the fever.

Knowing how frequently an epidemic of typhus follows in the wake of long continued destitution and starvation, and how much easier and better prevention is than cure, great praise is due to the Medical Officer of the Privy Council, Mr. Simon, for the promptitude he displayed in at once causing the medical inspection of the cotton districts in the time of their severe distress, and advising the adoption of measures which previous experience had shown in other cases were calculated to avert the frequent consequences of famine—overcrowding and destitution. That no severe outbreak of fever has occurred is to be thankfully attributed to the adoption of these measures. The anticipation of an epidemic of typhus was clearly warranted under the circumstances. The sole unquestionable proof that an epidemic was impending—namely, its subsequent occurrence—has, fortunately, been denied us on this occasion. We sincerely hope, however, that the utility of our efforts to avert epidemic disease may always admit of a similar negative demonstration.

Does one attack of typhus afford protection against a second, or, in other words, can one have typhus more than once? To this question it may be answered that, as a rule, a person does not have typhus more than once, any more than he has small-pox or measles more than once. One attack affords protection against a second; but although this is admitted as practically true, still there are exceptions to this, as to every other rule. Dr. Murchison tells us that he has himself had two distinct attacks of typhus fever; but in engaging nurses and attendants for typhus patients, especially if there are many cases, as in a hospital, it is always preferable to choose such as are already seasoned by an attack of fever. Dr. Murchison remarks that he had been unable to discover any instance of a nurse at the London Fever Hospital having had typhus twice, although some had been there for many years, whereas fresh nurses, during an epidemic, who had not had the disease before, were almost certain to be attacked.

Presenting a strong contrast to typhus, typhoid fever occurs quite independent of overcrowding or destitution, although the bad ventilation generally incident on overcrowding may increase the liability to contagion by favouring a concentration of the poison, and preventing its dilution by the atmosphere. It is much more common in Paddington, Belgravia, and the thinly-populated and more wealthy districts of the metropolis than in those quarters where typhus so predominates; it prevails in the outskirts of London, and is the form of fever usually met with in country districts. New comers to an infected locality are more liable to be attacked than the older inhabitants; the system gets gradually accustomed to the poison, and requires in time a larger dose probably to take effect.

The question whether typhoid fever be contagious has been argued at great length, and answered in different manners by different writers—some asserting contagion to be one of its most important characteristics, and others denying it to be contagious at all. We are inclined to conclude, with Dr. Murchison, who discusses the question at some length, that it is communicable in a limited degree, but it is impossible, in many instances, to discover any source of contagion. As this is a question of some interest, a short summary of the arguments which have been used to prove its contagious nature may not be out of place. When one individual is attacked, a series of cases often follow in the same house or locality; this may be explicable by the contagion theory of its transmissibility, or it may, on the other hand, be argued that the peculiar conditions of the locality which sufficed to produce one case are equally capable of producing a second, and a series of cases; and it seems that a great number of persons in the same locality are often attacked simultaneously. This occurs not unfrequently, and often gives rise to a suspicion of poisoning; and moreover when the cases occur in succession one after another, it is not the attendants or those who are mostly with the sick person that are always the first attacked; that is to say, there is no relation between the degree of exposure to contagion and the order of attack. It has been said that typhoid is communicated to the nurses and other attendants on the sick in private cases, but as the nurse and attendants live with the patient in the infected locality they are necessarily exposed to the same influences as those which produced the fever in the original case, and, as before stated, it is known that new comers into a tainted place are much more easily affected than the older inhabitants. In hospital practice the nurses rarely suffer from typhoid fever; the number of cases of typhoid fever among the nurses at the Fever Hospital is very small in comparison with those who suffer from typhus. Still cases do occur which only admit of explanation on the supposition of its contagious nature. Dr. Murchison relates the particulars of four cases where, undoubtedly, the fever was communicated by contagion. The most conclusive proof of its contagion is that it is often transported, by a person infected in one district to a locality where it was previously unknown, and from which it spreads as from a centre. The following cases narrated by Dr. Murchison will serve as an example of this kind of evidence. "In 1858, a servant ill of pythogenic fever was removed from Windsor to her home at Cippenham, four miles distant. Three weeks afterwards her father and sister took the disease, although no other cases had occurred at Cippenham. Another girl, ill of the fever, was removed to Bray, some miles distant. Shortly after her two sisters took the fever, although it was stated that no other cases had occurred at Bray previously. . . . Pythogenic fever broke out in a family living in an isolated country house on the top of a hill in France. Three nurses were called in to tend the sick. All three took the fever, and all three communicated it to their own families residing in a village at a long distance from the source of infection." Several examples of a similar kind are reported by Trousseau, in his *Clinique Médicale.*

Although in some instances propagated by contagion, it is equally true that many cases arise spontaneously, where all proof of contagion is absent; and it is now admitted by the great majority of writers that cases of spontaneous origin are quite as common as those where the source of contagion can be traced. The emanations from sewers and putrefying animal matter are regarded by many as a source of fever, and especially as a cause of typhoid fever; in fact the outbreak of typhoid fever in any locality at once excites suspicion that something must be wrong in the drainage; that, from some cause or other, there is an accumulation of night-soil denied its proper outlet, or some leakage in the system of pipes which has allowed it to permeate the soil and diffuse its exhalations in the neighbourhood, or that the water used for drinking purposes has been polluted by the admixture of some fæcal matter. In the case of the Westminster fever (1848), the Metropolitan Sanitary Commission gave it as their decided opinion that that epidemic arose from the bad state of the sewers and drains of the precinct, and especially from the foul condition of the large sewer; it was found that the disease followed very exactly in its course the line of a foul and neglected sewer, in which fæcal matter had been accumulating for years without any exit, and which communicated by direct openings with the drains of all the houses in which the fever occurred. The two following cases are selected from a series given by Dr. Murchison. They serve to illustrate the nature of the causes which produce the fever in as succinct a manner as is compatible with clearness.

"Towards the end of 1838, an epidemic of enteric fever desolated the Commune of Prades, in the Department of Ariège. Of the 750 inhabitants, 310 were attacked, and 95 died. The cause of the epidemic was traced to a stagnant pool, which was the receptacle of dead animals and of all the sewage of the district. The outbreak was preceded by damp, warm weather. Three times the pestilence returned, and always when the wind was blowing over the infected water (p. 439). During 1857, six policeman were admitted into the London Fever Hospital, from the Peckham police station, with enteric fever; three in June, one in July, one in August, and one in September. On inquiry, it was stated that there was no defect in the drainage of the building, and that the water-closets opened into the drains, and were well trapped. The men, however, affirmed that they had often complained of dreadful odours in the room in which they sat. I accordingly applied to the officer of health for the district to have the building carefully examined. The result was the discovery that one water-closet on the ground floor, emptied itself not into the main drain, with which it had no connexion whatever, but into an old well, immediately underneath the passage adjoining the room in question. Here an accumulation of upwards of ten feet of soil had been going on for years, and the top of the well was merely covered by the flagstones of the passage. The cesspool was removed, and the fever ceased." (p. 443).

The following cases show that typhoid is sometimes produced by drinking the contaminated water to which allusion has already been made.

"In the latter part of 1859, Bedford was the seat of a severe outbreak of enteric fever, although before this it had been 'the autumnal habit of the town to suffer from it.' On investigation, it was found that the distribution of the fever did not follow the ramifications of the sewers, nor did it appear to depend on the escape of cesspool air into the houses, but there was every reason to believe that it was due to fæcal matter soaking into the wells, from the numerous cesspools of the town. The water from these wells was found to contain a large quantity of decaying animal matter, evidently derived from the sources alluded to." (p. 446).

At Richmond-terrace, Clifton, a crescent consisting of thirty-four houses, intestinal fever broke out in thirteen of them. The houses in which it broke out were far apart in the terrace, and there was little or no intercourse between their inmates. On investigation, it turned out that these thirteen houses were supplied with water by the same well, and that this water was tainted, as was evident from the taste and smell, by sewage; the other houses were supplied with water from another source, and were unvisited by any outbreak of the fever. It has been maintained by some, that when the disease arises as it has been often found to do under similar circumstances to the last mentioned cases, it is then produced only by the presence of typhoid evacuations in the sewers and the drinking water, and that the smallest quantity of an alvine evacuation derived from a typhoid patient, if present in the liquid mentioned, would be sufficient to produce the fever. But then how can we account for the first case which occurs in a locality far distant from any site known to be affected with the disease (in some country district for example), without admitting its origin *de novo*. Of course in large towns, where there are always a few cases present, it is impossible to prove positively that no typhoid evacuation is present in the sewage; but to assume that it must be present because typhoid fever occurs seems to be somewhat circular ratiocination, and makes as great a demand on our faith as to admit the origin of the poison *de novo* under certain conditions.

The rate of mortality of typhoid, calculated from above eighteen thousand cases, is eight and a half per cent., being very nearly the same as in typhus.

In uncomplicated cases, the mean duration of typhus is about fourteen days, while the ordinary duration of typhoid fever is from three to four weeks, and when once convalescence has fairly commenced in typhoid, the recovery takes much longer to complete than in typhus.

One of the most common complications of typhus is bronchitis, with more or less hypostatic congestion of the lungs, which not unfrequently proves fatal. True pneumonia, on the contrary, is comparatively rare. A peculiar inflammation of the subcutaneous cellular tissue of the lower extremities, resembling phlegmasia dolens, is said to have been very common in some epidemics of typhus; of late years it has not been so often met with. Gangrene, commencing in the toes and extending upwards, causing sometimes the destruction of the entire foot, and rendering amputation necessary, sometimes occurs. Inflammatory swellings of the parotid and sub-maxillary glands have been frequently observed in some epidemics of typhus; although not by any means necessarily fatal, they increase the gravity of the prognosis considerably. Pneumonia is more common in typhoid than in typhus; phlegmasia dolens seems common to both fevers, but peritonitis is a complication peculiar to typhoid, and its most common cause is perforation of the bowels.

We may here mention that the disease known as infantile remittent fever—a febrile state of the system, accompanied with gastric and intestinal symptoms, not unfrequently met with in children, is now, by most writers, considered identical with typhoid fever, and the majority of cases of "gastric fever" are probably of the same nature.

Our space will not permit us entering into the particulars of the distinctive characters of relapsing or famine fever. We shall only note that a careful review of the symptoms fully justifies its separation from typhus on the one hand and typhoid on the other. As its name, famine fever, intimates, it is especially due to want of food and scarcity of food; for a series of bad harvests, or otherwise, has generally been the forerunner of an epidemic. It has not been nearly so frequent in its appearance as typhus. It formed the majority of cases of the great epidemic of fever of 1816–18, which began in Ireland, and subsequently extended to this country. There was another epidemic in 1826, not so extensive as the above mentioned, and at the end of this epidemic typhus fever became very prevalent. In 1842 there was also an epidemic, chiefly in Scotland, and again in 1848. At the end of this latter epidemic, typhus also prevailed to a great extent, bearing the same relation, in this respect, to the epidemic of 1842 as that of 1826 to that of 1818. Since the epidemic of 1848, it has been gradually disappearing from this country; there was a considerable increase in the number of cases of relapsing fever in London in 1851; since that time they have become very rare. Dr. Murchison says he has reason to believe that, since 1855, not a single case has been observed, either in London or Glasgow. It is generally admitted to be contagious; the laws which regulate its transmission are similar to those which have been mentioned for typhus, and it also can be generated *de novo*. Dr. Murchison is inclined to believe that starvation, in contra-distinction to overcrowding, is the great exciting cause of relapsing fever. The most peculiar feature of the disease is the occurrence of ophthalmia during the period of convalescence. It is said to be met with most frequently among cases who have had insufficient nourishment during that period. It is often serious in its ultimate effect upon the vision, and is generally very tedious in its course. Its frequent occurrence would seem also to intimate that deficient nutrition was intimately related to the fever in question.

The rate of mortality of relapsing fever, calculated from the result of over 14,000 cases, is 4·75 per cent., or 1 in 21; much less fatal than typhus and typhoid.

The last chapter of Dr. Murchison's book is occupied with a discussion of the various arguments which have been brought forward on the relative advantages of treating fever among the patients of a general hospital, or isolating them in a special hospital, devoted solely to cases of fever. His conclusions are strongly in favour of special Fever Hospitals; for he proves, from statistical observations, first, that the rate of mortality among the patients treated in the London Fever Hospital is not greater than among the cases of fever treated in general hospitals; and secondly, that the danger of spreading the fever is infinitely less.

During the first six months of 1862, 1107 cases of true typhus were under treatment in the London Fever Hospital, of which 232 died, or the mortality was 20·95 per cent. In the same period, 343 cases of typhus were under treatment in six of the general hospitals of the metropolis, of which number 80 died, or 23·32 per cent. The 1080 (1107–27) cases admitted into the Fever Hospital communicated the disease to 27 persons, of whom 8 died. In other words, only 1 person took the fever for every 40 admitted, and 1 died for every 135. But the 272 cases admitted into the six general hospitals communicated the disease to 71 persons, of whom 21 died, or 1 person caught the fever for every 3·8 cases admitted, and 1 life was lost for every 12·9 cases admitted; and Dr. Murchison inquires what would have been the result if there had been no Fever Hospital, and if the 1080 cases admitted into it had been distributed among the general hospitals, in addition to the few hundreds which were actually treated by them? The objection that among a number of patients with typhus the fever poison becomes more concentrated, and hence more liable to carry infection, than where a few cases are sprinkled in a ward amongst other diseases, is easily obviated by having large, well ventilated wards in a fever hospital, and never allowing any approach to overcrowding. Dr. Murchison remarks that if 2000 cubic feet of space be allowed to each patient, and if there be thorough ventilation, there need be no more concentration of the poison in a fever hospital than in a general hospital with a sprinkling of fever cases. These remarks apply, of course, to typhus only. It would seem that cases of typhoid may be admitted into a general hospital with impunity; but in proportion to the number of cases of typhus treated the danger of the disease spreading is much less on the plan of isolation than in that of mixing.

We would now commend this great systematic work to the attention of our readers. It fills up a much felt hiatus in the medical literature of this country, and will be welcomed by every physician and by all who take any interest in the public health.

